# The most important question in family approach: the potential of the resolve item of the family APGAR in family medicine

**DOI:** 10.1186/s12930-016-0028-9

**Published:** 2016-05-05

**Authors:** Hiroaki Takenaka, Nobutaro Ban

**Affiliations:** Takenaka Clinic, Osaka, Japan; Department of General Medicine/Family & Community Medicine, Nagoya University Graduate School of Medicine, Nagoya, Japan

**Keywords:** Family, Family research, Family members

## Abstract

**Background:**

We aimed to clarify what aspects of family function are measured by the Family APGAR by examining its correlations with the fourth edition of the Family Adaptability and Cohesion Evaluation Scale at Kwansei Gakuin (FACESKG IV). Furthermore, we sought to confirm the usefulness of the Family APGAR in general practice.

**Methods:**

We recruited 250 patients (aged 13–76 years) from the general medicine outpatient clinic in a Japanese hospital between July 1999 and February 2000. We employed a cross-sectional design and administered the Family APGAR and the FACESKG IV-16 (i.e., the short version). The scores on the questionnaires were compared using correlation and multiple regression analyses. We then analyzed relationships between the questionnaires and family issues measures using Chi square, Mann–Whitney U, and logistic regression analyses.

**Results:**

The Family APGAR partially evaluates the Cohesion dimension of family functioning as measured by the FACESKG IV-16. Furthermore, we could measure family disengagement using the resolve and partnership items of the Family APGAR. Family dysfunction (excessive or impoverished Adaptability or Cohesion) was not related to the presence of family issues. Nevertheless, there was a significant relationship between scores on the Resolve item and the family issues measure (χ^2^ = 6.305, p = 0.043).

**Conclusions:**

The Family APGAR, especially the Resolve item, has the potential for use in treating patients with family issues. Interventions could be developed according to the simple Family APGAR responses.

**Electronic supplementary material:**

The online version of this article (doi:10.1186/s12930-016-0028-9) contains supplementary material, which is available to authorized users.

## Background

Practicing family medicine relies on sufficient understanding of the biopsychosocial aspects of patients. In this context, family is considered the most important aspect of patients’ social environments. However, currently, family approaches to medicine are not widespread among Japanese family physicians. This is likely because family medicine places excessive emphasis on the value of the family conference; more specifically, Japanese family physicians must treat 10–20 patients per hour in outpatient clinics, which means that they must spend roughly 3–6 min per patient. For this reason, many physicians hesitate to hold family conferences, which require considerable time and skill. A promising means of circumventing this problem, however, would be to utilize questionnaires.

The Family APGAR has frequently been utilized as a tool for assessing family function (Smilkstein [1]). Developed in 1978, it is a 5-item questionnaire (with each item rated on a 3-point scale) measuring five constructs: “Adaptability,” “Partnership,” “Growth,” “Affection,” and “Resolve.” Investigations of the reliability and validity of the questionnaire led to its revision by Smilkstein, Ashworth, and Montano in 1982 [2]. Because the Family APGAR consists of only five questions, it is relatively easy and quick to administer; this has made it the preferred choice for evaluating family function in primary care and general medicine settings. However, Gardner et al. [3] pointed out that it is somewhat unclear what the scale items actually measure. Nevertheless, the test remains widely (and perhaps blindly) utilized. In Japan, numerous university-based general practitioners and family nursing practices use the Family APGAR to educate students.

The Family Adaptability and Cohesion Evaluation Scale (FACES; Olson et al. [4]) is another fairly simple instrument for assessing family function. The FACES is a companion measure for the Circumplex model of marital and family systems (hereafter known as the Circumplex model; Olson et al. [5]), one of the most widely used yet highly controversial models of family function. This model emphasizes that optimal family functioning is a balance between two dimensions: “Cohesion” and “Adaptability.” Cohesion is defined as the degree of emotional bonding family members have with one another. Excessive closeness results in “enmeshment”—families exhibit extreme amounts of emotional closeness and may be dependent on, and highly reactive to, one another. Additionally, high levels of family loyalty and consensus are required and there is little tolerance for private space or relationships outside the family. Excessive separateness, in contrast, causes “disengagement,” where families exhibit little emotional closeness and instead remain focused on individual experiences and activities. Furthermore, such families have limited commitment to family interests, and members are often unable to turn to one another for emotional or practical support or assistance. For Cohesion, balance would refer to “separated” or “connected” families, where both individual and group interests are valued [6]. Adaptability, on the other hand, is defined as the ability of a marital or family system to change its power structure, role relationships, and relationship rules in response to situational and developmental stress. Poor Adaptability leads to “rigidity,” wherein the family or couple relationship is unable to shift or evolve in response to change, whether that arising internally through individual members’ development or that imposed by the environment. Excessive Adaptability, on the other hand, results in “chaos,” with family members unable to create shared agreements that govern their actions and inter-relationships, and thus providing no firm base on which they can stand. In between these two extremes lie the balanced options of “flexible” or “structured” families, where the balance between rigidity and chaos is negotiated from the strong base of shared understanding of rules and roles within the relationship [6].

Foulke et al. [7] administered the Family APGAR and FACES II (the second version of FACES) to 140 families and found that the Family APGAR correlated with the relevant Circumplex model dimensions of the FACES II (Cohesion, r = 0.70; Adaptability, r = 0.59). However, in another study with 66 families, no association was found between the Family APGAR and the FACES II (Clover et al. [8]). One possible explanation for these conflicting results is that the dimensions of the FACES II are curvilinear. In other words, moderate levels of Adaptability and Cohesion are optimal, but too much or too little is dysfunctional under normal circumstances. This accords with the properties of the Circumplex model, wherein the avoidance of extremes for either dimension is emphasized. However, evidence for curvilinearity in the FACES, FACES II, and FACES III has not yet been demonstrated.

One Japanese research group was successful in identifying the curvilinearity of their original scale. Tatsuki developed the FACES at Kwansei Gakuin (FACESKG) series, which considers the cultural and social milieu of Japan [9]; the curvilinearity of the scale dimensions was identified in the 32-item fourth edition (FACESKG IV-32) by Tatsuki [10]. A shorter adaptation of the FACESKG IV-32 was also created, called the FACESKG IV-16; this is a 16-item Thurstone scale questionnaire [8] that is suited for use in a general medicine setting because it is succinct and easy to administer. The scale results are based on the sum of the score of each question multiplied by a coefficient appropriate for the content. However, Japanese clinics often comprise only a few staff members, such as a physician, nurse, and clerk, which means that they would have little time to complete a questionnaire. Indeed, even questionnaires with few questions such as the FACESKG IV-16 would not be easy to administer in daily work in Japan.

Thus, the current study had three objectives. The first was to clarify what aspects of family function are measured by the Family APGAR, by examining the correlations between the Family APGAR and the FACESKG IV-16, for which linearity and curvilinearity, respectively, have been established in the Japanese population. In Japanese family practice, previous studies have noted that physicians do not like administering the full Family APGAR, despite the fact that it comprises only five questions. Therefore, we wanted to identify the particularly effective questions for analyzing family dysfunction, thereby enabling the Family APGAR to be used in daily clinical practice more conveniently. As such, we analyzed the relations of each item score of the Family APGAR with the FACESKG IV-16 in addition to the total Family APGAR score.

It is generally believed that family issues occur in response to family dysfunction (i.e., excessive or impoverished functioning). As such, the second objective was to confirm the correlations between family dysfunction and family issues, and to identify the particularly important aspects of family function by investigating the correlations between FACESKG IV-16 scores and the family issues measured.

The third and final objective was to confirm the validity of the Family APGAR as a basis for helping families cope with family issues; for this purpose, we examined the correlation between the Family APGAR and the family issues measure.

We defined “family dysfunction” as having a score of 2 or more on the absolute values of the Cohesion and Adaptability item scores of the FACESKG IV-16, and a score of <8 on the total Family APGAR score (with <4 being indicative of severe dysfunction). We defined “family issues” as the suffering that participants experienced as a result of their family.

## Methods

### Design

The study design was cross-sectional and employed two questionnaires (the Family APGAR and the FACESKG-IV) and one original question assessing family issues.

### Setting and participants

The present study was conducted at the outpatient clinic of a university hospital in Japan (Department of Primary Care Medicine, Kawasaki Medical School, Kurashiki City, Okayama, Japan) between July 1999 and February 2000. Thirteen clinicians administered the questionnaires to their patients. Study participants completed the Family APGAR (translated into Japanese from the original English version) and FACESKG IV-16. In order to evaluate family issues, we devised an original question: “Do you have any worries about your family? If you do, please tell us about them. You are free to decline to answer.” We excluded patients who declined to participate, were unable to understand the scale items, did not provide answers to all items on both questionnaires, were experiencing acute disease, or reported uncomfortable feelings while responding to the scale items.

### Procedures

We explained the contents of the study and enrolled patients who agreed to participate. Written informed consent was then obtained from all participants. Patients completed the questionnaire while waiting at the billing department after their medical examinations. Completed questionnaires were then brought to the front desk of the outpatient clinic.

### Statistical analyses

We employed correlation and multiple regression analyses (the step-down procedure) to compare Family APGAR measures with scores on the FACESKG IV-16. We then analyzed the relationships between FACESKG IV-16, the Family APGAR, and family issues by using a Chi square test, a Mann–Whitney U test, and a logistic regression analysis because setting family issues as the dependent variable required a binomial distribution whereas the independent variables utilized a curvilinear model. Data were analyzed using SPSS^®^ version 11.0.

### Ethics

Written informed consent was obtained from all subjects. We applied for ethical approval to the Institutional Ethical Review Board of the Kawasaki Medical School through the professor in charge; however, the board deemed it exempt from ethical approval. We then submitted it to the Ethical Review Board of the Osaka Society of Family Practice, who approved the study protocol.

## Results

Participants were 311 patients, of whom 250 (80.4 %) gave complete responses. Participants (gender: 120 male, 126 female, 4 unknown) ranged in age from 13 to 76 years (*M* = 49.2, *SD* = 13.2) and had an average of 3.5 (*SD* = 1.6) family members. One hundred six participants had mental disorders, 45 had hypertension, 45 had hyperlipidemia, and 38 had diabetes mellitus. Seventy-six patients (30.4 %) reported having some family issues. Specifically, family issues included health problems with their family member (*n* = 24), family lifecycle issues (e.g., family death, aging; *n* = 17), problems with family dynamics (*n* = 6), substance abuse or addiction (e.g., alcohol, gambling; *n* = 5), work-related problems (e.g., unemployment, irregularity of work; *n* = 6), economic problems (*n* = 3), and unknown problems (i.e., the participant did not want to answer; *n* = 20). Note that five participants reported two issues, which is why there is a discrepancy in the number of participants for family issues (with *n* = 76 reporting family issues overall but *n* = 81 when summing the number of participants reporting specific issues).

**Fig. 1:**
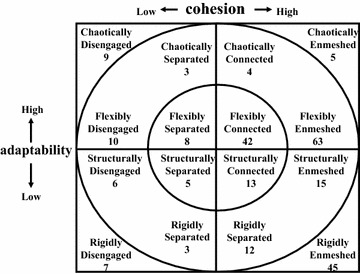
Family functioning on the circumplex model

**Fig. 2:**
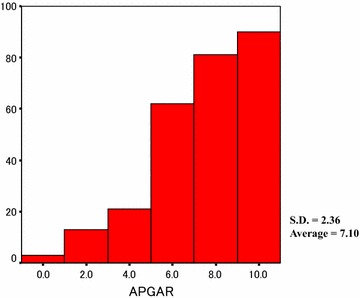
The distribution of the total family APGAR score

Figure 1 shows the family function of the participants according to the Circumplex model. There were 68 (27.2 %) balanced families, 116 (46.4 %) mid-range families, and 66 (26.4 %) unbalanced (i.e., dysfunctional) families. Figure 2 shows the distribution in Family APGAR scores; according to this measure, family function can be categorized as “good” (scores from 7 to 10), “moderate dysfunction” (score from 4 to 6), or “severe dysfunction” (score from 0 to 3). In the present study, 171 (63.3 %) patients reported good family function, 77 (28.5 %) reported moderate dysfunction, and 22 (8.1 %) reported severe dysfunction. Thus, the results indicated that the definition of family dysfunction differs substantially between the FACESKG IV-16 and the Family APGAR.

### What aspects of family function does the Family APGAR measure?

**Fig. 3:**
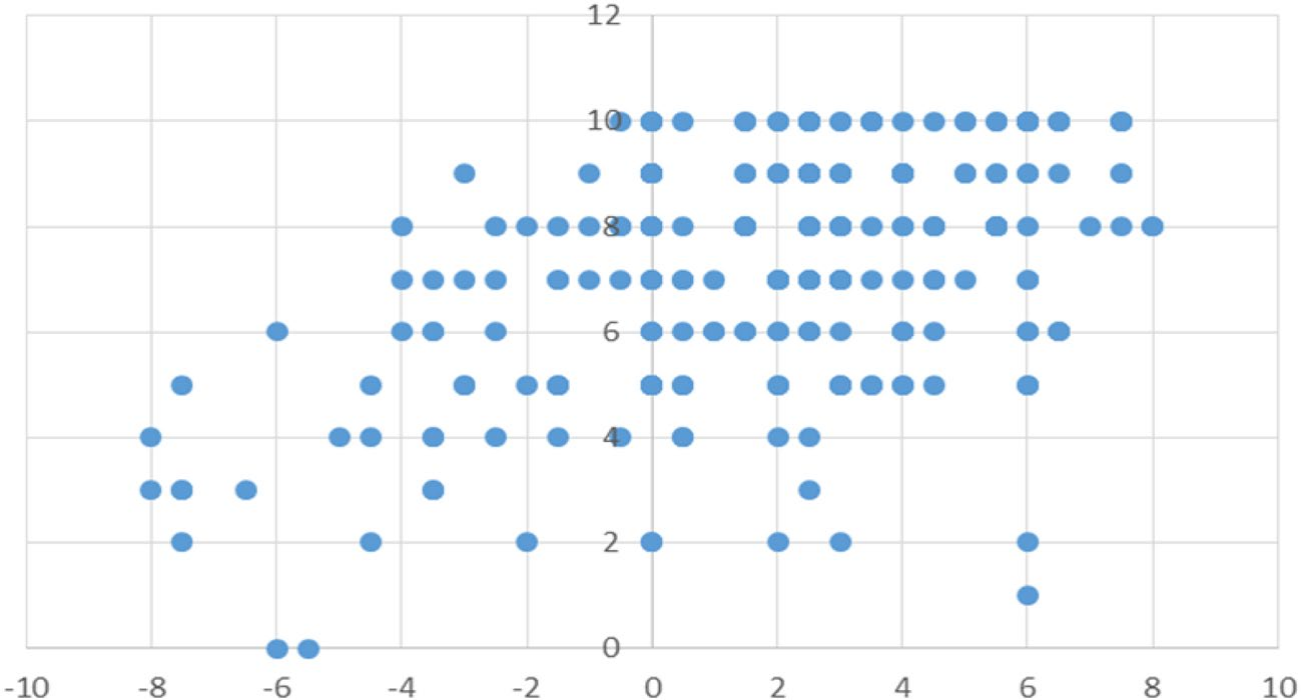
The scatter diagram between the Cohesion score on the FACESKG IV-16 and the total Family APGAR score

**Fig. 4:**
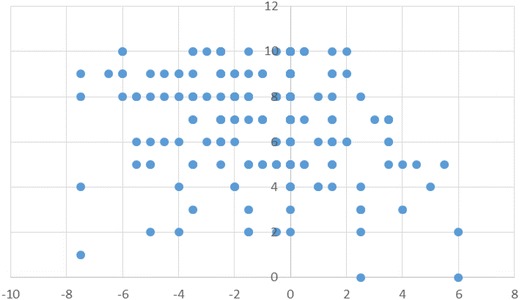
The scatter diagram between the Adaptability score on the FACESKG IV-16 and the total Family APGAR score

**Fig. 5:**
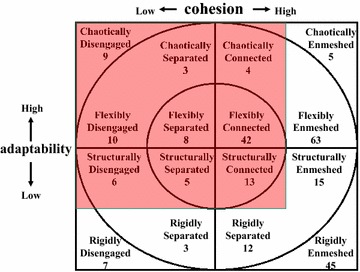
The range that the family APGAR measures

Figure 3 shows a scatter diagram between the Cohesion score on the FACESKG IV-16 and the total Family APGAR score. Additional file 1: Table S1 shows results for the correlation analysis for the Cohesion score on the FACESKG IV-16, the total Family APGAR score, and each Family APGAR item score. The total Family APGAR score and each item score were significantly correlated with the FACESKG IV-16 Cohesion score (p < 0.01). The largest correlation was with the Resolve score of the Family APGAR (*r* = 0.549). Figure 4 shows the scatter diagram between the Adaptability score on the FACESKG IV-16 and the total Family APGAR score, while Additional file 2: Table S2 shows the results for the correlation analysis of the FACESKG IV-16 Adaptability score with Family APGAR scores. The total Family APGAR score and the Adaptability, Partnership, Growth, and Affection item scores were all significantly and negatively correlated with the FACESKG IV-16 Adaptability score (p < 0.05); the largest correlation was with the Partnership item (*r* = −0.210). Scores on the Resolve item were not significantly correlated with the FACESKG IV-16 Adaptability score, but were significantly correlated with the squared values of the Adaptability score (p < 0.01). These results indicate that the Resolve item of the Family APGAR measures family disengagement and chaos, and partially measures rigidity (Fig. 5). However, the Family APGAR could not measure enmeshment or fully measure rigidity.

Additional file 3: Table S3 shows the results of the multiple regression analysis using the step-down procedure. In the best-fit model, the predictor variable was the FACESKG IV-16 Cohesion score while the outcome variables were the Partnership and Resolve item scores from the Family APGAR (Adjusted R^2^ = 0.322, p < 0.01). The regression equation was as follows: Cohesion score = 0.191 × Partnership score + 0.472 × Resolve score − 2.982. The regression analysis showed that Adaptability was not significantly explained by the Family APGAR items. Thus, family cohesion can be assessed utilizing only two questions—the Partnership and Resolve items of the Family APGAR—but family adaptability cannot be assessed using the Family APGAR.

Then, we analyzed the sensitivity and specificity of the total Family APGAR score in predicting the FACESKG IV-16. The sensitivity of the total Family APGAR score for predicting the Cohesion score of the FACESKG IV-16 was 24.1 % and the specificity was 70.3 %. The sensitivity of the total Family APGAR score in predicting the Adaptability score of the FACESKG IV-16 was 31.8 % and the specificity was 77.4 %. These results indicate that the Family APGAR partially measures family cohesion and the Resolve and Partnership items could be used to capture family disengagement; however, the sensitivity of the total Family APGAR score for family adaptability was only 24.1 %. Therefore, we might utilize the total Family APGAR score only to exclude the possibility of family disengagement.

### The correlations between family dysfunction and family issues

We concluded our investigation by analyzing the relationships between family dysfunction (as measured by the FACESKG IV-16) and family issues utilizing a Chi square test. Interestingly, neither dysfunctional Cohesion (excessive or impoverished) nor dysfunctional Adaptability was significantly related to family issues. This indicates that family dysfunction (i.e., excessive or impoverished Cohesion and Adaptability) does not always occur in the presence of family issues.

### The validity of the Family APGAR in measuring ability to cope with family issues

**Fig. 6:**
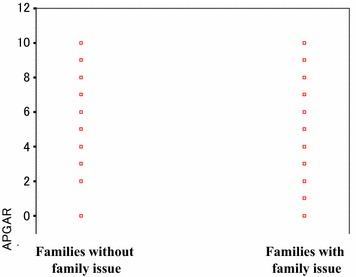
Scatter diagram between the total Family APGAR score of families with issue and without one

Figure 6 shows a scattergram of the relationships between the total Family APGAR scores and family issues. The results of a Mann–Whitney U (ranking) test showed that the total Family APGAR scores of families with family issues were significantly lower than were the scores for families without family issues (p < 0.05). Next, we analyzed the relationships between each item of the Family APGAR and the family issues measure by utilizing a Chi square test. The results for the Adaptability (χ^2^ = 0.946, p = 0.623), Partnership (χ^2^ = 2.314, p = 0.314), Growth (χ^2^ = 2.467, p = 0.291), and Affection (χ^2^ = 3.076, p = 0.215) items were all non-significant. However, there was a significant relationship between scores on the Resolve item and the family issues measure (χ^2^ = 6.305, p = 0.043). A further Mann–Whitney U test revealed that patients with family issues had significantly lower scores on the Resolve item than did patients with no family issues (p < 0.05).

Differences by gender, age, or disease were not observed in any of the analyses.

## Discussion

We found that the Family APGAR partially measured family cohesion. Furthermore, family issues did not always occur in the presence of family dysfunction (excessive or impoverished Cohesion and Adaptability). This latter result is important because in the past it was generally believed that family issues occur in response to family dysfunction, while our results indicate that improving family function might not help solving family issues. It is possible that excessive or impoverished family cohesion and adaptability are not dysfunctions but rather are coping styles for dealing with family issues. In that case, changing family dynamics might weaken the family’s style of coping with their issues. Thus, family physicians should avoid blindly attempting to change family dynamics.

We found that the Family APGAR, especially the Resolve item, has the potential to become a tool for measuring family function, at least in terms of family issues. Most importantly, the Resolve item was able to distinguish patients with family issues from those with no such issues. This finding may be important to general practitioners who operate busy clinics or are inexperienced with the family approach. Applying the simple Resolve item—“Are you satisfied with the way you and your family share time together?”—might be the most efficient way to assess whether patients have family issues. For example, in a daily clinical setting, a family physician might ask a patient “Do you have any worries about your family?” If the patient affirms this, the physician might go on to ask the Resolve item. The Resolve item may be useful for identifying patients for whom a family conference—wherein the physician assembles family members and encourages them to communicate without employing special techniques—would be beneficial, and can be the first step in implementing a family approach for physicians who might normally avoid it. As such, the Resolve item of the Family APGAR may be a highly useful tool in family medicine.

We note several limitations to our study. First, the cross-sectional design did not allow us to examine changes in family function over time. We also excluded participants with acute disease because their conditions precluded their answering the questionnaire. In addition, this study was conducted in Japan in a specialized environment wherein physicians must treat 10–20 patients per hour, and relied heavily on Japanese cultural values concerning spending time with one’s family. Further study will be required to confirm whether the results generalize across cultures.

A further limitation concerns the publication of our results, which has taken a considerable amount of time and effort because of a struggle to translate our findings into English while also performing our clinical duties. Considering the extensive gap between data collection and publication, it is possible that the Japanese family structure and social context differ nowadays compared to when the study was first conducted. In order to translate our ideas successfully, we required repetitive checking and translation by a native English speaker. However, this process was highly costly. Furthermore, there were few specialists available to us who were familiar with general medicine and family approaches and who were native English speakers. Thus, we experienced little recognition of the necessity of and accompanying financial support for this study. In addition, all authors of this study were both researchers and practicing physicians, which made it difficult to complete the manuscript. It must be noted that our study remains important despite the time taken to publish it. We are releasing these results because of their importance; however, we do intend to perform follow-up studies to further validate them.

## Conclusions

The Family APGAR partially evaluates the Cohesion dimension of family functioning as measured by the FACESKG IV-16; furthermore, its Resolve and Partnership items are able to capture family disengagement. In addition, family dysfunction (excessive or impoverished Adaptability or Cohesion) was not related to the presence of family issues. Nevertheless, the Family APGAR, especially the Resolve item, has some potential for treating patients with family issues. Thus, interventions could be developed according to the simple Family APGAR responses.

## Additional files

10.1186/s12930-016-0028-9 Correlation analysis between cohesion score of FACESKG IV and total family APGAR score.
10.1186/s12930-016-0028-9 Correlation analysis between adaptability score of FACESKG IV and the total family APGAR score.
10.1186/s12930-016-0028-9 Multiple regression analysis (the dependent valuable was the cohesion score of FACESKG IV).

## Abbreviations

FACES: the family adaptability and cohesion evaluation scale
FACESKG: the family adaptability and cohesion evaluation scale-Kwansei Gakuin
FACESKG IV: the family adaptability and cohesion evaluation scale-Kwansei Gakuin version 4
