# Supplementary figures and images for: The most important question in family approach: the potential of the resolve item of the family APGAR in family medicine
Source: Asia Pac Fam Med. 2016 May 5;15:3. doi: 10.1186/s12930-016-0028-9 (PMC4858843; doi:10.1186/s12930-016-0028-9)

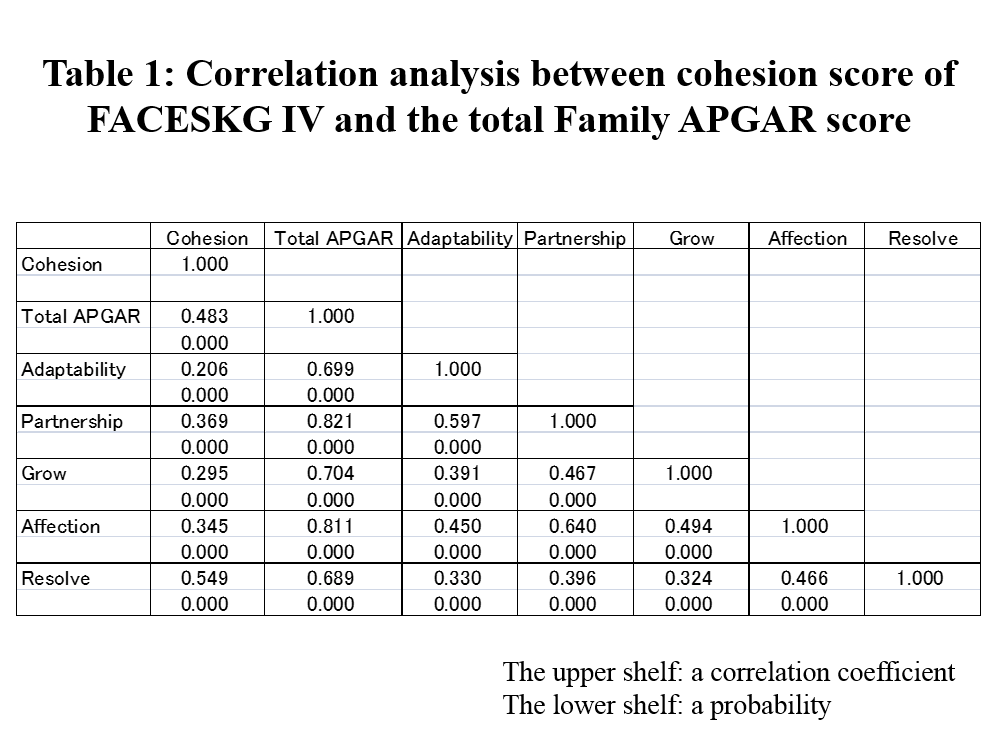

Supplement: Supplementary file 1 — 10.1186/s12930-016-0028-9 Correlation analysis between cohesion score of FACESKG IV and total family APGAR score. [file 12930_2016_28_MOESM1_ESM.doc]

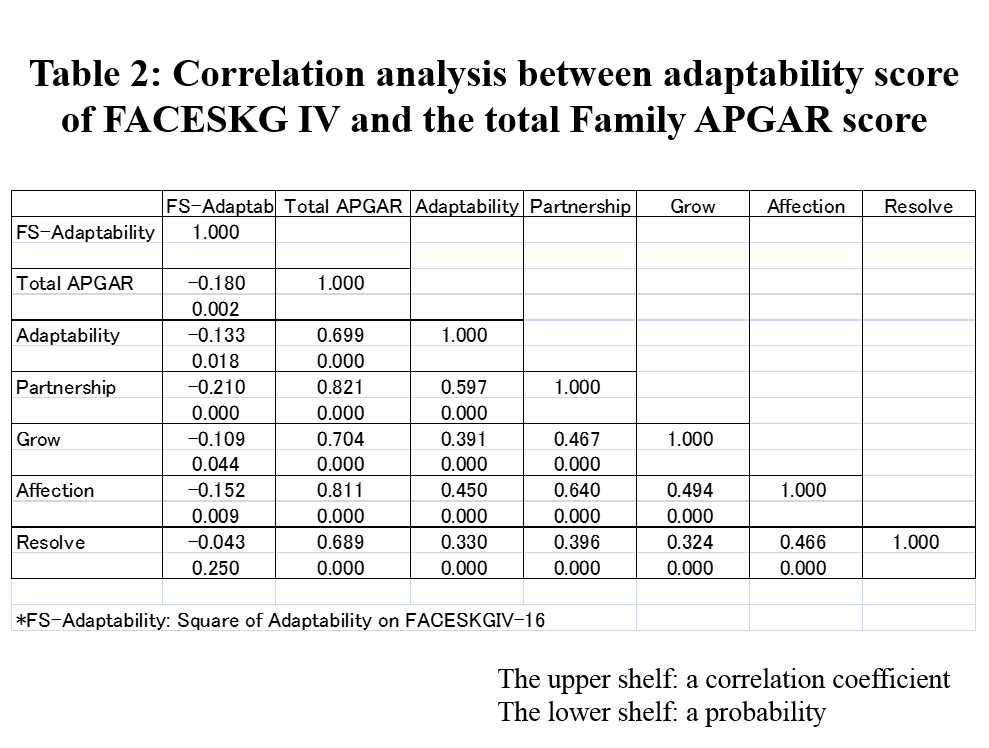

Supplement: Supplementary file 2 — 10.1186/s12930-016-0028-9 Correlation analysis between adaptability score of FACESKG IV and the total family APGAR score. [file 12930_2016_28_MOESM2_ESM.doc]

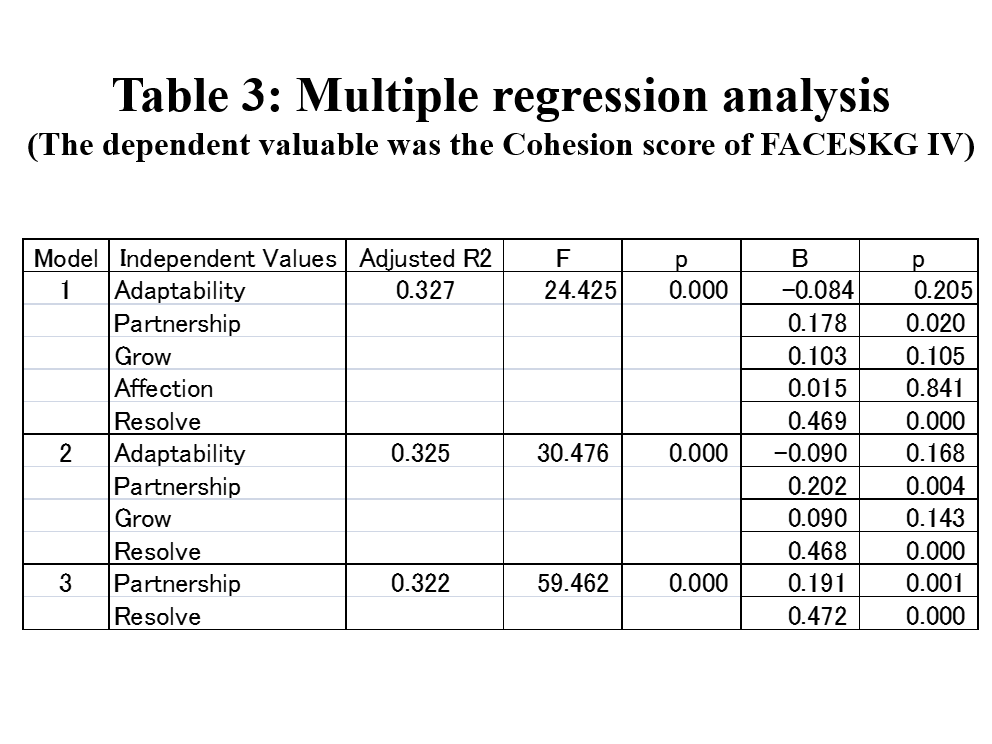

Supplement: Supplementary file 3 — 10.1186/s12930-016-0028-9 Multiple regression analysis (the dependent valuable was the cohesion score of FACESKG IV). [file 12930_2016_28_MOESM3_ESM.doc]
